# In Silico Identification of Potential Inhibitor Against a Fungal Histone Deacetylase, RPD3 from *Magnaporthe Oryzae*

**DOI:** 10.3390/molecules24112075

**Published:** 2019-05-31

**Authors:** Gnanendra Shanmugam, Taehyeon Kim, Junhyun Jeon

**Affiliations:** Department of Biotechnology, College of Life and Applied Sciences, Yeungnam University, Gyeongsan, Gyeongbuk 38541, Korea; gnani.science@gmail.com (G.S.); puppispp@gmail.com (T.K.)

**Keywords:** histone deacetylases, *Magnaporthe oryzae*, virtual screening, molecular docking, MoRPD3

## Abstract

Histone acetylation and deacetylation play an essential role in the epigenetic regulation of gene expression. Histone deacetylases (HDAC) are a group of zinc-binding metalloenzymes that catalyze the removal of acetyl moieties from lysine residues from histone tails. These enzymes are well known for their wide spread biological effects in eukaryotes. In rice blast fungus, *Magnaporthe oryzae*, MoRPD3 (an ortholog of *Saccharomyces cerevisiae Rpd3*) was shown to be required for growth and development. Thus in this study, the class I HDAC, MoRpd3 is considered as a potential drug target, and its 3D structure was modelled and validated. Based on the model, a total of 1880 compounds were virtually screened (molecular docking) against MoRpd3 and the activities of the compounds were assessed by docking scores. The in silico screening suggested that [2-[[4-(2-methoxyethyl) phenoxy] methyl] phenyl] boronic acid (−8.7 kcal/mol) and [4-[[4-(2-methoxyethyl) phenoxy] methyl] phenyl] boronic acid (−8.5 kcal/mol) are effective in comparison to trichostatin A (−7.9 kcal/mol), a well-known general HDAC inhibitor. The in vitro studies for inhibition of appressorium formation by [2-[[4-(2-methoxyethyl) phenoxy] methyl] phenyl] boronic acid has resulted in the maximum inhibition at lower concentrations (1 μM), while the trichostatin A exhibited similar levels of inhibition at 1.5 μM. These findings thus suggest that 3D quantitative structure activity relationship studies on [2-[[4-(2-methoxyethyl) phenoxy] methyl] phenyl] boronic acid compound can further guide the design of more potential and specific HDAC inhibitors.

## 1. Introduction

During the last few decades, extensive work on how epigenetic factors such as histone acetylation contribute to the regulation of gene expression has been carried out. Such histone acetylation is a dynamic and reversible process that is mediated by the concerted activity of histone acetyltransferases (HAT) and histone deacetylases (HDAC) [1]. These enzymes are well known for their wide-spread biological and developmental effects in higher organisms [2,3,4]. For this reason, much effort has been put in using in silico drug designing strategies to screen for novel potential inhibitors of HDACs, potentially targeting cancer cells [5,6,7,8,9,10]. Histone deacetylases are a group of metal-dependent enzymes that catalyze the removal of acetyl moieties from lysine residues from histone tails and are being considered as important targets [11]. Based on the sequence similarity and enzymatic function, the HDACs are grouped into four classes such as class I, II, III, and IV [12]. Among these, class I, II, and IV are considered as classical HDACs with a zinc dependent active site and are inhibited by a well-known HDAC inhibitor trichostatin A (TSA), whereas the class III enzymes possess NAD^+^-dependent active sites, which are not affected by TSA [1]. In fact, the TSA was discovered initially as an antifungal antibiotic against *Trichophyton* sp. and later was identified as an inhibitor of HDACs that can manipulate the histone acetylation levels [13,14,15]. 

The classical fungal HDACs have been characterized and divided into two classes: Class I or Reduced Potassium Dependency 3 (RPD3)-type HDACs (including RPD3, HOS1, and HOS2) and the class II or HDA1-type HDACs (HDA1) [16]. RpdA and HosA, from *Aspergillus nidulans*, were the first identified HDACs in class I RPD3-type enzymes [17]. Later, several studies on HDACs in filamentous fungi have reported that class I HDACs are involved in regulating many important genes for fungal development and pathogenicity [18,19,20]. In *A. nidulans*, it had been shown that *RPD3* is essential for the fungus [21]. Recently, we also found that deletion of the *RPD3* gene (*MoRPD3*) in the rice blast fungus, *Magnaporthe oryzae*, renders the fungus inviable (unpublished data). This line of evidences suggests that RPD3 could be a promising target for identification and development of new agrochemicals that can effectively control fungal diseases in crop plants.

*M. oryzae* is an ascomycete fungus that is a causal agent of the rice blast disease [22]. Infection usually starts with dissemination of asexual spores, called conidia. The conidium that lands on the leaf surface can germinate in the presence of water, and then develops a dome-shaped infection-specific structure known as an appressorium, with which the fungus breaches the cuticular layer of plants to gain access to the plant tissues [23,24,25,26,27,28]. Therefore, the appressorium formation is considered as a critical step for successful infection. Here, in this study, in silico drug designing strategies were applied for the identification of novel HDAC inhibitor through virtual screening. The binding affinities of best ten compounds against MoRPD3 in comparison to well-known HDAC inhibitor, trichostatin A, are reported. Furthermore, two novel inhibitors identified from our virtual screening were tested for their ability to inhibit fungal growth and appresorium formation.

## 2. Results and Discussion

Recently we found that MoRPD3 (an ortholog of *Saccharomyces cerevisiae RPD3* in *M. oryzae*) that belongs to putative class I HDAC gene is required for vegetative growth and appressorium formation, a key process in *M. oryzae* infection. In this present study, we have applied structure-based drug designing strategies to identify the novel HDAC inhibitor in comparison to trichostatin A (TAS) through virtual screening. To validate the inhibitory activity of the compounds, we have tested the effects of the compounds on fungal growth and appressorium formation. 

### 2.1. Target-Template Alignment and Homology Modelling 

**Figure 1:**
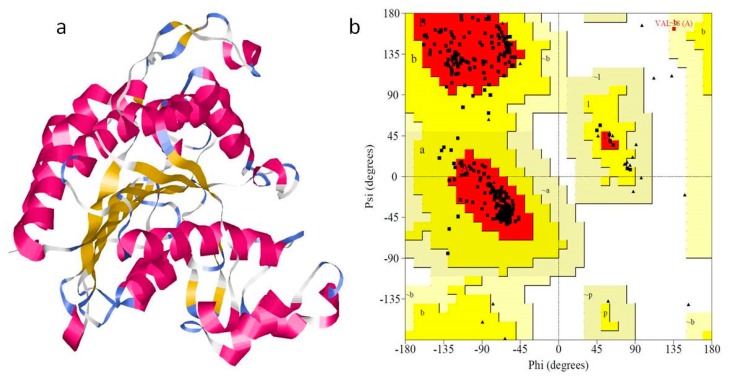
Theoretical model of MoRPD3 structure and model validation with PROCHECK. (**a**) The 3D structure of built protein in ribbon representation; helices are shown in magenta and sheets in yellow (**b**) model validation by Ramachandran plot.

The histone deacetylase RPD3 protein sequence (Uniprot ID: G4N3Q0) from *Magnaporthe oryzae* was used to run a BLASTP (Basic Local Alignment Search Tool for Protein) search against the protein databank (PDB). As a result, we obtained the x-ray crystal structures of human Hdac2 in complex with vorinostat (PDBID:4lxz_A chain) as homologous protein that shares sequence identity of 67.86%. Few studies have demonstrated that sequence identity higher than 25% between two proteins are similar in 3D structures [29,30]. Hence, the 3D structure of human Hdac2 in complex with vorinostat was considered as a suitable template for homology modeling. In modeler 9v9, the template–target sequence alignment file and template structure co-ordinates files are used to generate the 3D model (homology model) of MoRPD3 (Figure 1a) and considered for further analysis. 

### 2.2. Model Validation

The Structural Analysis and Verification Server (SAVES) [31] of UCLA-DOE Lab providing the quality evaluation tools such as PROCHECK, ERRAT, and Verify 3D were used to assess the quality of the modelled MoRPD3 structure. The Ramachandran plot (RC plot) exploring the stereo-chemical parameters such as phi and psi angles were determined by using PROCHECK [32]; Verify 3D [33] was used to determine the 1D–3D structure compatibility and the regions of the modelled structure that can be rejected at the 95% and 99% confidence intervals were predicted through ERRAT programs [34]. The RC plot of template, human Hdac2 in complex with vorinostat (PDBID:4lxz_A chain) exhibited 91.7% residues in most favored regions and 0% residues in disallowed regions. Similarly, the RC plot of the generated model revealed that the built model is the best as it exhibited a higher number of residues (93%) in the most favorable regions, while a lower number of residues (0.3) are observed in additionally the allowed region and 0% in the disallowed region (Figure 1b). Furthermore, the measured quality factor values of ERRAT plot (Figure S1a) and Verify 3D (Figure S1b) supported that the built model is relevant, reliable, and of good quality (Table 1). 

### 2.3. In Silico Drug Designing

**Figure 2:**
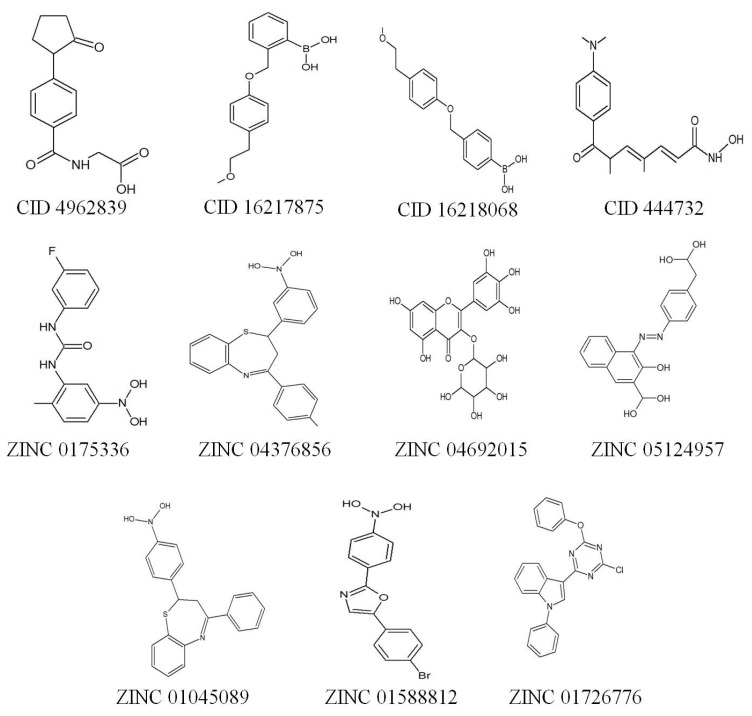
List of best 10 compounds (2D structures) that exhibited better binding affinity with MoRPD3 in comparison with trichostatin A (CID 444732).

The binding sites of the modelled protein were predicted by using the CASTp server. From the predicted binding sites, the sites with larger volumes were used for further virtual screening (by molecular docking interactions) with the compounds from the ZINC database [35] and Pubchem database [36]. The top 10 compounds (Figure 2) that exhibited better binding affinities against MoRPD3 proteins were reported in Table 2. The drug-like properties that satisfy the Lipinski’s rule of five [37], which is considered to be essential for rational drug design, were determined for all the top 10 compounds. It was observed that all the compounds did not violate Lipinski’s rule of five (Table S1), i.e. ≤5 hydrogen bond donors, ≤10 hydrogen bond acceptors, <500 Dal of molecular weight, <5 partition coefficient (log P), < 10 rotatable bonds, and topological polar surface area (TPSA) <140.

**Figure 3:**
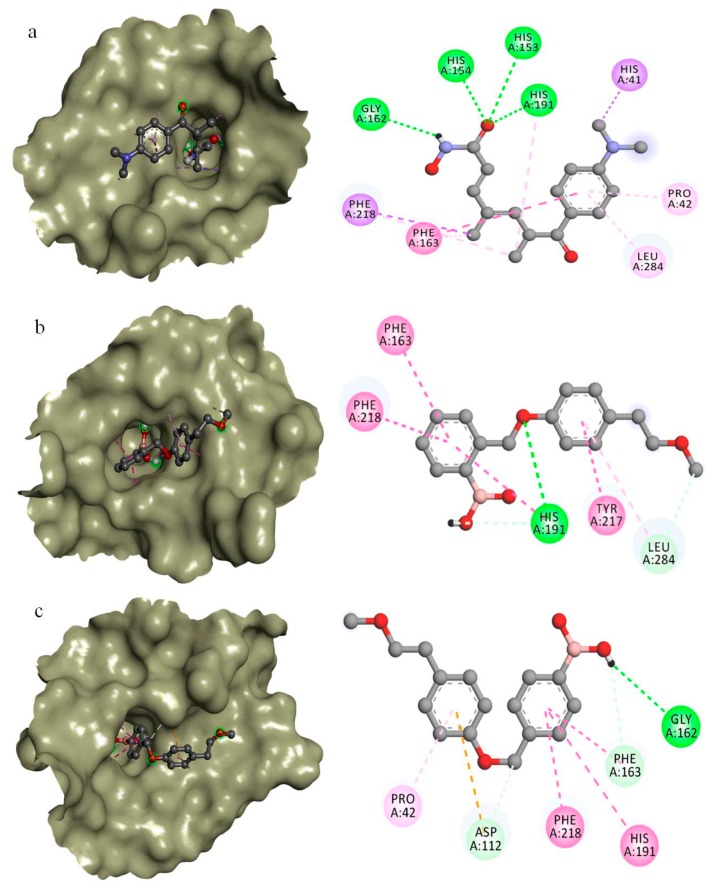
The docking complex and interactions of best docked compounds with modelled MoRPD3 from *M. oryzae*. (**a**) Trichostatin A (binding affinity: −7.0 kcal/mol) (**b**) CID16217875 (binding affinity: −8.7 kcal/mol) (**c**) CID16218068 (binding affinity: −8.5 kcal/mol).

The binding affinities and the molecular basis of interactions of the two best compounds docked within the predicted binding sites of MoRPD3 along with Trichostatin A are explored (Table 3). The binding affinity and interactions of top 10 compounds against MoRPD3 are given in Supplementary Table S2. From the docking results, it is evident that all the top 10 compounds exhibited better binding energy against MoRPD3 (Table 2). The docking interactions of trichostatin A (CID444732) is favoured by H-bonds with His153, His154, Gly162, and His191, while non-bonded hydrophobic interactions are favoured by His41, Pro42, Phe218, and Leu284 (Figure 3a). The binding affinity of trichostatin A is −7.0 kcal/mol, where as the two identified compounds; CID16217875 and CID16218068 exhibited binding affinities of −8.7 and −8.5 kcal/mol. The interactions of CID16217875 [2-[[4-(2-methoxyethyl) phenoxy] methyl] phenyl] boronic acid is favored by H-bond interactions with His191 and Leu284, while non-bonded hydrophobic interactions are supported by Phe163, Try217, and Phe218 (Figure 3b). In case of CID16218068 ([2-[[4-(2-methoxyethyl) phenoxy] methyl] phenyl] boronic acid), the H-bonded interactions are supported by Asp112, Gly162, and Phe163. The hydrophobic non-bonded interactions are formed with Pro42, His191, and Phe218 (Figure 3c). Generally, the non-bonded interaction is involved in making the protein-ligand complex more stable and establishing non-bonded force (Vander Waal’s) to make the ligands achieve its stable conformation for better activity [38].

Taken together, the present docking studies of two novel HDAC inhibitors of MoRPD3 from *M. oryzae* implies that the Phe163 and His 191 in the binding pocket are crucial for interactions. Therefore, these prioritized drug target and drug compounds may play a pivotal role in the development of new HDAC inhibitors against *M. oryzae* infections.

### 2.4. In Vitro Studies for Appressorium Formation Inhibition

**Figure 4:**
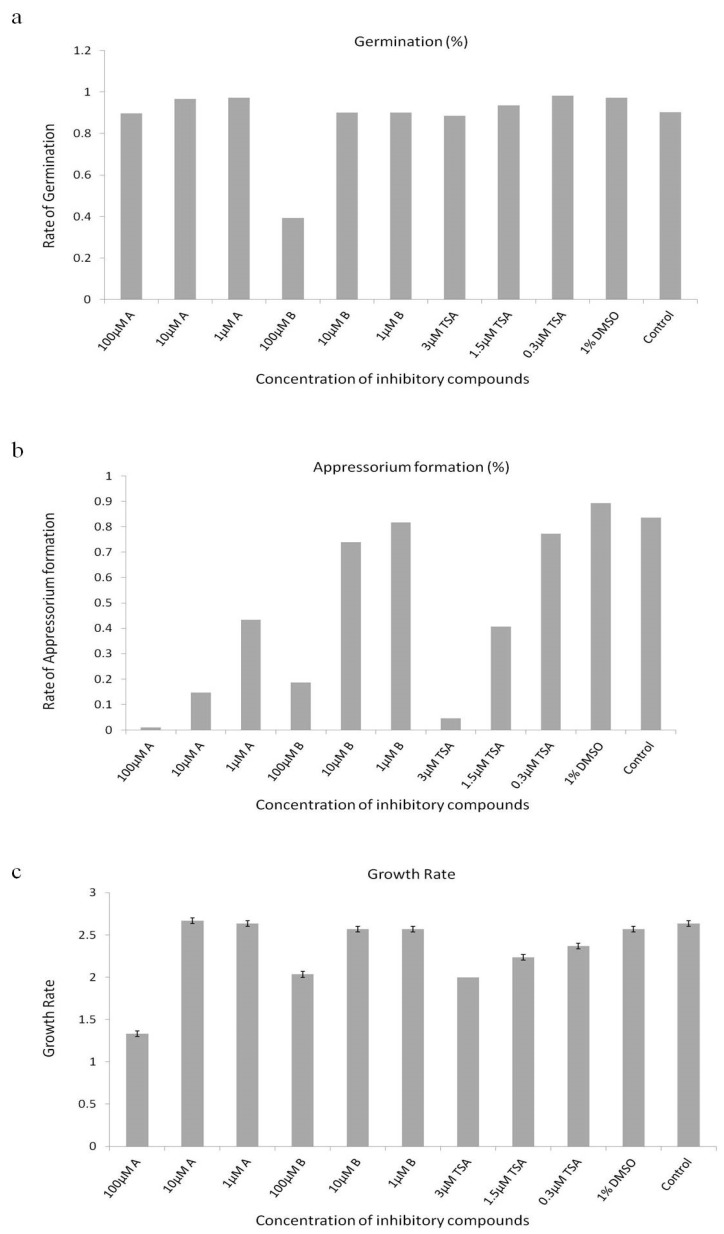
Effects of two identified HDAC inhibitors (in this study) in comparison with trichostatin A and DMSO. (**a**) Rate of germination (%) (**b**) appressorium formation (%) (**c**) growth rate of *M. oryzae* KJ201.

The inhibitory effects of novel HDAC inhibitors on fungal growth and appressorium formation of *M. oryzae* were tested by supplementing plate culture with compounds or by placing the conidia on hydrophobic coverslips in the presence of varying concentrations of compounds. Surprisingly, the addition of the compounds to the media did not greatly affect the radial growth of the fungus (Figure 4a). In the control experiment without compounds, the rate of germination and appresorium formation were 89% and 85%, respectively. In the presence of 0.3 µm to 3 µm of TSA, the rate of appresorium formation significantly decreased. Similarly, the other HDAC inhibitor, [2-[[4-(2-methoxyethyl) phenoxy] methyl] phenyl] boronic acid (compound A) resulted in the maximum inhibition at lower concentrations (1 μM). The other inhibitor, [4-[[4-(2-methoxyethyl) phenoxy]methyl] phenyl] boronic acid (compound B), however, exhibited low inhibition activity on growth rate, rate of germination, and appresorium formation in comparison to trichostatin A and compound A. Dimethyl Sulfoxide (DMSO), the solvent used to dissolve the chemical compounds did not show any effects on the germination and appressorium formations (Figure 4a,b). In a similar study, Izawa et al. [23] reported that the rate of appressorium formation and germination significantly reduced in the presence of 0.5 and 1 μg/mL of TSA. They also reported that the other two HDAC inhibitors (sodium butyrate and Trichostatin C) showed significant appressorium formation inhibition activity. In another study, Caracuel-Rios and Talbot showed that TSA affects the appressorium formation in another plant pathogenic fungus, *Colletotrichum lagenarium* [39,40]. Evidently, these results suggest that proper regulation of histone acetylation may play a vital role in infection-related development in plant pathogenic fungi. 

Our data and previous works suggest that MoRPD3 plays an important role in appressorium formations, and that its inhibition by TSA and the other two compounds may lead to a hypothesis that these compounds significantly impair MoRPD3 functions. However, the fact that the two compounds identified in this study was not able to completely inhibit the appressorium formation suggests either that inhibition of MoRPD3 is incomplete or that HDAC activity of remaining HDACs such as MoHDA1 (MGG_01706), MoHOS2 (MGG_01633), and MoHOS3 (MGG_06043) might be contributing to appressorium formations as well. In conclusion, the 3D quantitative structure activity relationship studies regarding [2-[[4-(2-methoxyethyl) phenoxy] methyl] phenyl] boronic acid (compound A) would further guide the design of more potential and specific HDAC inhibitors targeting all the four classical histone deacetylases of *M. oryzae*. 

## 3. Materials and Methods

### 3.1. Sequence Analysis for Potential Templates

The histone deacetylase RPD3 protein sequence (Uniprot ID: G4N3Q0) from *Magnaporthe oryzae* was retrieved from UniProtKB database [41]. Using BLASTP (basic local alignment search tool) [42] similarity search tool against PDB database [43], the most homologous sequence was obtained and considered as a potential template for homology modeling and its respective atomic coordinate file from PDB was obtained for homology modeling. The sequence alignment and alignment errors were refined by using the ClustalW program [44] as the sequence alignment reflects the quality of the homology models.

### 3.2. Homology Modeling

Using the homology modeling tool, Modeler 9v9 [45], the homology models of MoRPD3 were built by employing the target–template sequence alignment files. A total of 5 3D models were built from the starting structure of the templates by satisfying the spatial restraints through random generation [46]. Among the generated models, the least root mean square deviation (RMSD) value in comparison with the template structure was considered for selecting the best model and its energy was minimized through 20 steps of steepest descent and conjugate gradient by using GROningen Molecular Simulation (GROMOS) force field of Swiss-PDB viewer [47], and final energy-minimized model was used for further analysis.

### 3.3. Model Validation

The stereo-chemical parameter of the energy-minimized model was considered to evaluate the quality of the generated models. The phi and psi angles representing the stereo-chemical parameters of the model through PROCHECK [32], the compatibility of a generated 3D structure with its own amino acid sequence through Verify3D [33], and the regions of the modelled structure that can be rejected at the 95% and 99% confidence intervals through ERRAT [34] were determined at SAVES [31].

### 3.4. Structure-Based Virtual Screening

The binding pockets in the modelled structure were identified by submitting to the CASTp (Computed Atlas of Surface Topology of proteins) server. For virtual screening, the compounds from the ZINC database [35] and pubchem database [36] along with the well known HDAC inhibitor trichostatin A were used. The docking analysis was performed by using AutoDock vina [48] by setting the docking grid size to 57 Å × 56 Å × 58 Å, which encompassed the entire MoRPD3 structure. 

### 3.5. Docking Interactions

The docking interactions that envisaged the binding affinities of the compounds within the predicted binding pocket amino acids of the modelled MoRPD3 were analyzed by using discovery studio visualize [49], that clearly revealed the H-bond and non-bond interactions. 

### 3.6. In Vitro Studies for Appressorium Formation Inhibition

The mycelia of *M. oryzae* KJ201 grown on oatmeal agar plates at 25 °C for 10 days and the conidiation was induced under the light while the plates were unsealed. The conidia were suspended in distilled water containing various concentrations of TSA and 2 identified compounds. The 2 compounds were dissolved in DMSO. Then 40 µL of the mixture was placed on hydrophobic glass slides and incubated at 25 °C for 8 h in a moistened box [50]. After incubation, the rate of germination and appressorium formation ratio was calculated both in the presence and absence of drugs. In addition, the effect of DMSO was calculated separately.

## Figures and Tables

**Table 1:** The measured quality factor values along with Ramachandran plot residue distributions for the modelled proteins.

S.No.	Model	Amino Acids Residues (%) in Ramachandran Plot (PROCHECK)				Verify 3D (%)	ERRAT
		MFA	GAR	AAR	DAR		
1.	MoRPD3 Model	93.0	6.7	0.3	0.0	91.21	93.25
2.	4LXZ Template	91.7	8.2	0.1	0.0	94.11	95.83

MFA-Most favoured region; GAR-Generously allowed region; AAR—Additionally allowed region; DAR-Disallowed region.

**Table 2:** List of top 10 best compounds exhibited better binding affinities compared to Trichostatin A (CID444732).

S.No	Compound ID	IUPAC Name	Binding Affinity (kcal/mol)
1	CID444732	(2*E*,4*E*,6*R*)-7-[4-(dimethylamino)phenyl]-*N*-hydroxy-4,6-dimethyl-7-oxohepta-2,4-dienamide (Trichostatin A)	−7.0
2	CID4962839	2-[[4-(2-oxopyrrolidin-1-yl)benzoyl] amino] acetic acid	−7.2
3	CID16217875	[2-[[4-(2-methoxyethyl) phenoxy] methyl] phenyl] boronic acid	−8.7
4	CID16218068	[4-[[4-(2-methoxyethyl) phenoxy]methyl] phenyl] boronic acid	−8.5
5	ZINC01753336	1-(3-fluorophenyl)-3-(2-methyl-5-nitro-phenyl)urea	−7.9
6	ZINC04376856	4-(4-methylphenyl)-2-(3-nitrophenyl)-2,3-dihydro-1,5-benzothiazepine	−8.0
7	ZINC04692015	5,7-dihydroxy-3-((2s,3r,4r,5r,6s)-3,4,5-trihydroxy-6-methyl-tetrahydro-pyran-2-yloxy)-2-(3,4,5-trihydroxy-phenyl)-1-benzopyran-4-one	−7.6
8	ZINC05124957	4-[4-(carboxymethyl)phenyl]azo-3-hydroxy-naphthalene-2-carboxylic	−7.1
9	ZINC01045089	2-(4-nitrophenyl)-4-phenyl-2,3-dihydro-1,5-benzothiazepine	−8.1
10	ZINC01588812	5-(4-Bromo-phenyl)-2-(4-nitro-phenyl)-oxazole	−7.3
11	ZINC1726776	3-(4-chloro-6-phenoxy-s-triazin-2-yl)-1-phenyl-indole	−7.8

**Table 3:** Amino acids in the binding pockets of the Modelled MoRPD3 structure favouring H-bond and non-bonded interactions with two best docked compounds and HDAC inhibitor, trichostatin A (CID444732).

CID444732	CID16217875	CID16218068
His41 (Pi-Sigma)	-	-
Pro42 (Pi-Alkyl)	-	Pro42 (Pi-Alkyl)
-	-	# Asp112 * (Pi-Anion)
His153 *	-	-
His154 *	-	-
Gly162 *	-	Gly162 *
Phe163 (Pi-Pi)	Phe163 (Pi-Pi-stacked)	# Phe163 * (Pi-Pi-stacked)
# His191 * (Pi-Alkyl)	# His191 * (Pi-Pi)	His191 (Pi-Pi-stacked)
-	Tyr217 (Pi-Pi-stacked)	-
Phe218 (Pi-Alkyl)	Phe218 (Pi-Pi-stacked)	Phe218 (Pi-Pi-stacked)
Leu284 (Pi-Alkyl)	# Leu284 * (Pi-Alkyl)	-
−7.0 kCal/mol	−8.7 kcal/mol	−8.5 kcal/mol

* Residues involved in H-bond interactions; # * Residues involved in H-bond and non-bonded interactions. The other residues are involved in non-bonded interactions. The types of hydrophobic interactions are provided in braces. Binding affinities (kcal/mol) are provided respectively.

## Supplementary Materials

The following are available online, Figure S1: Verify 3D plot showing the 1D–3D structure compatibility of the modelled MoRPD3 from *Magnaporthe oryzae*, Figure S2: ERRAT Plot showing the generated model as good high resolution as the regions of the modeled structure that can be rejected at the 95% and 99% of confidence is low. Table S1: The predicted molecular properties confined to the druglike properties (based on Lipinski’s rule of five)., Table S2: Amino acids in the binding pockets of the Modelled MoRPD3 structure favouring H-bond and non-bonded interactions with top 10 docked compounds.
