# Supplementary material for: In Silico Identification of Potential Inhibitor Against a Fungal Histone Deacetylase, RPD3 from Magnaporthe Oryzae
Source: Molecules. 2019 May 31;24(11):2075. doi: 10.3390/molecules24112075 (PMC6600661; doi:10.3390/molecules24112075)
Supplement: Supplementary file 1 [file molecules-24-02075-s001.pdf]

Supplementary Data

In-silico **identification of potential inhibitor against a fungal histone deacetylase, RPD3 from** ***Magnaporthe oryzae***

Gnanendra Shanmugam, Taehyeon Kim and Junhyun Jeon*

Department of Biotechnology, College of Life and Applied Sciences, Yeungnam University, Gyeongsan, Gyeongbuk, 38541, Korea

* Correspondence to Junhyun Jeon

Phone: +82 2 810-3030

E-mail: jjeon@yu.ac.kr

Supplementary Figure S1

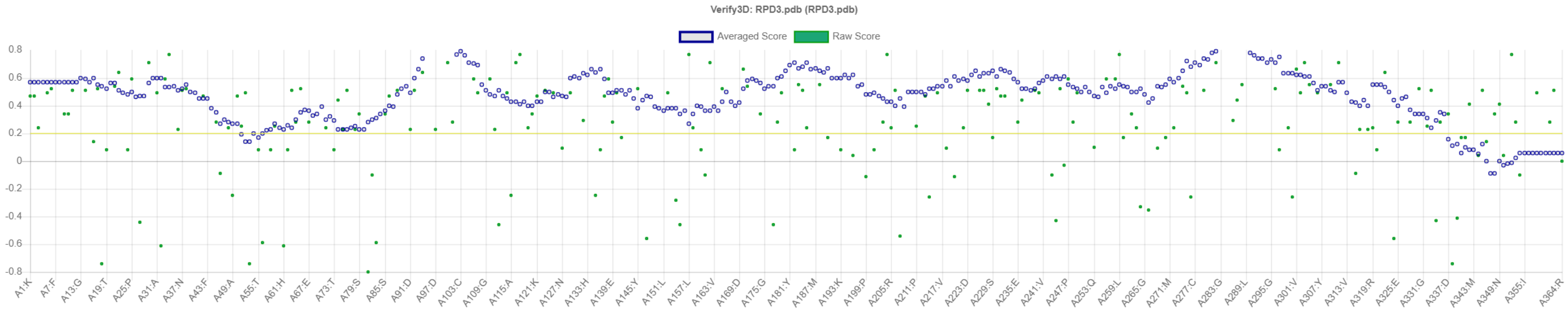

Verify 3D plot showing the 1D–3D structure compatibility of the modelled MoRPD3 from *Magnaporthe oryzae*.

Supplementary Figure S2

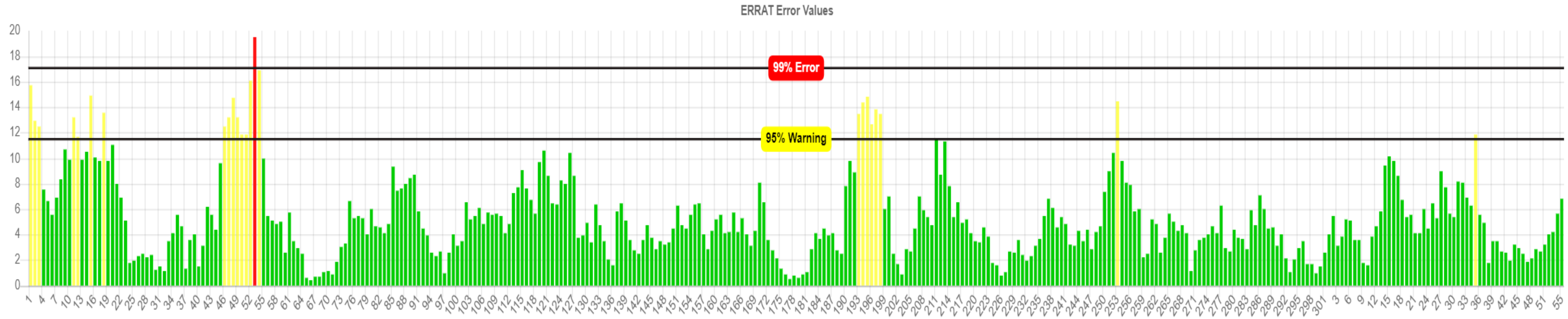

ERRAT Plot showing the generated model as good high resolution as the regions of the modeled structure that can be rejected at the 95% and 99% of confidence is low.

Supplementary Tables

Supplementary Table S1. The predicted molecular properties confined to the druglike properties (based on Lipinski's rule of five).

Compounds Pubchem ID	Molinspiration server predictions								
	miLogP	TPSA	natoms	Mol.Wt	nON	nOHNH	nviol	nrotb	volume
CID444732	2.68	69.64	22	302.37	5	2	0	6	293.12
CID4962839	-1.17	86.71	19	262.26	6	2	0	4	230.56
CID16217875	2.80	58.92	21	286.14	4	2	0	7	294.46
CID16218068	2.75	58.92	21	286.14	4	2	0	7	295.46
ZINC01753336	3.62	86.95	21	289.27	6	2	0	3	244.07
ZINC04376856	4.98	58.19	27	374.46	4	0	1	3	330.93
ZINC04692015	0.35	110.50	33	464.38	9	8	2	3	371.96
ZINC05124957	1.03	125.21	26	348.31	7	1	0	5	291.88
ZINC01045089	4.86	58.19	26	360.44	4	0	1	3	314.37
ZINC01588812	4.83	71.86	21	345.15	5	0	0	3	245.49
ZINC1726776	5.98	52.84	29	398.85	5	0	1	4	337.68

The molecular properties for the compounds are predicted at molinspiration server to define the compounds druglikness. miLogP-Molinspiration LogP (Octanol-water partition coefficient); TPSA- Total polar surface area (drug transport properties); natoms-Number of atoms; Mol.Wt (g/mol)- Molecular weight, nON- number of hydrogen bond acceptors; nOHHN- number of hydrogen bond donors; nviolations-Number of Lipinski's rule of five parameters violations; nrotb-Number of Rotatable Bonds (molecular flexibility).

Supplementary Table S2. Amino acids in the binding pockets of the Modelled MoRPD3 structure favouring H-bond and non-bonded interactions with top 10 docked compounds

CID 444732	CID 4962839	CID 16217875	CID 16218068	ZINC 01753336	ZINC 04376856	ZINC 04692015	ZINC 05124957	ZINC 01045089	ZINC 01588812	ZINC 1726776
His41 (Pi-Sigma)		-	-	-	-	-	-	-	-	-
Pro42 (Pi-Alkyl)		-	Pro42 (Pi-Alkyl)	-	Pro42 (Pi-Alkyl)	-	-	-	-	-
-	-	-	#Asp112* (Pi-Anion)	-	Asp112*	-	-	#Asp112* (Pi-Anion)	-	#Asp112* (Pi-Anion)
His153*	-	-	-	-	-	-	-	-	-	-
His154*	-	-	-	His154*	-	-	-	His154*	-	-
Gly162*	-	-	Gly162*	-	-	Gly162*	-	-	Gly162*	-
Phe163 (Pi-Pi)	Phe163 (Pi-Sigma)	Phe163 (Pi-Pi-Stack)	#Phe163* (Pi-Pi- Stack)	Phe163 (Pi-Pi-Stack)	Phe163 (Pi-Sigma)	Phe163 (Pi-Sigma)	Phe163 (Pi-Sigma)	Phe163 (Pi-Sigma)	Phe163 (Pi-Sigma)	Phe163 (Pi-Sigma)
#His191* (Pi-Alkyl)	-	#His191* (Pi-Pi)	His191 (Pi-Pi- Stack)	His191 (Pi-Pi-Stack)	His191 (Pi-Pi- Stack)	#His191* (Pi-Pi)	His191 (Pi-Pi- Stack)	His191 (Pi-Pi- Stack)	His191 (Pi-Pi- Stack)	His191 (Pi-Pi- Stack)
-	-	Tyr217 (Pi-Pi-Stack)	-	Tyr217 (Pi-Pi-Stack)	-	-	-	-	-	-
Phe218 (Pi-Alkyl)	Phe218 (Pi-Alkyl)	Phe218 (Pi-Pi-Stack)	Phe218 (Pi-Pi- Stack)	Phe218 (Pi-Pi-Stack)	Phe218 (Pi-Pi- Stack)	Phe218 (Pi-Pi- Stack)	Phe218 (Pi-Pi- Stack)	Phe218 (Pi-Pi- Stack)	Phe218 (Pi-Pi- Stack)	Phe218 (Pi-Pi- Stack)
Leu284 (Pi-Alkyl)	#Leu284* (Pi-Sigma)	#Leu284* (Pi-Alkyl)	-	Leu284 (Pi-Sigma)	Leu284 (Pi-Alkyl)	Leu284 (Pi-Alkyl)	Leu284 (Pi-Alkyl)	-	Leu284 (Pi-Alkyl)	Leu284 (Pi-Alkyl)
-	Gly285*	-	-	-	-	-	-	-	-	-
-	-	-	-	-	Tyr316 (Pi-Alkyl)	-	-	-	-	-
-7.0 kcal/mol	-7.2 kcal/mol	-8.5 kcal/mol	-8.7 kcal/mol	-7.9 kcal/mol	-8.0 kcal/mol	-7.6 kcal/mol	7.1 kcal/mol	-8.1 kcal/mol	-7.3 kcal/mol	-7.8 kcal/mol

* Residues involved in H-bond interactions; #*Residues involved in H-bond and non-bonded interactions. The other residues are involved in non-bonded interactions. The types of hydrophobic interactions are provided in braces. Binding affinities (kcal/mol) are provided respectively.
